# Real or Artificial? Intergroup Biases in Mind Perception in a Cross-Cultural Perspective

**DOI:** 10.1371/journal.pone.0137840

**Published:** 2015-09-11

**Authors:** Eva G. Krumhuber, Aleksandra Swiderska, Elena Tsankova, Shanmukh V. Kamble, Arvid Kappas

**Affiliations:** 1 Department of Experimental Psychology, University College London, London, United Kingdom; 2 Department of Psychology and Methods, Jacobs University Bremen, Bremen, Germany; 3 Department of Psychology, Karnatak University, Dharwad, India; Cardiff University, UNITED KINGDOM

## Abstract

Recent research suggests that attributions of aliveness and mental capacities to faces are influenced by social group membership. In this article, we investigated group related biases in mind perception in participants from a Western and Eastern culture, employing faces of varying ethnic groups. In Experiment 1, Caucasian faces that ranged on a continuum from real to artificial were evaluated by participants in the UK (in-group) and in India (out-group) on animacy, abilities to plan and to feel pain, and having a mind. Human features were found to be assigned to a greater extent to faces when these belonged to in-group members, whereas out-group faces had to appear more realistic in order to be perceived as human. When participants in India evaluated South Asian (in-group) and Caucasian (out-group) faces in Experiment 2, the results closely mirrored those of the first experiment. For both studies, ratings of out-group faces were significantly predicted by participants’ levels of ethnocultural empathy. The findings highlight the role of intergroup processes (i.e., in-group favoritism, out-group dehumanization) in the perception of human and mental qualities and point to ethnocultural empathy as an important factor in responses to out-groups.

## Introduction

One of the most fundamental interpersonal goals is to establish and maintain a meaningful connection with fellow humans. This can only be achieved once we detect another mind to connect with [1]. Hence, for social behavior to occur, it is crucial to attribute mental states both to the self and to other actors, and to consider their intentions, attitudes, beliefs, and emotions. Such ability has been referred to as Theory of Mind (ToM) [2] or mentalizing [3]. It is assumed that through inference or simulation of the content of people’s minds, we are able to understand their ongoing actions, predict future behavior, and coordinate it with our own [4]. Imputing others with an independent mind in fact constitutes the core of perceiving them as complete human beings with moral status [5]. Yet, however natural it is for us to expect that others have minds, those minds are after all inaccessible and their presence is ultimately a matter of perceptual processes. In this paper, we investigate how the perception of mind behind human faces varies with ethnic group membership and to what extent it is influenced by the perceiver’s empathic ability.

With recent advances in computer technology, an increasing attention is being devoted to how people perceive non-living entities, for example virtual agents, avatars, and robots [6,7]. Their visual appearance acts as an important cue to social perception and has an impact on the assumption of capabilities (e.g., [8,9]). Research has shown that people effortlessly distinguish between real and artificial faces [10,11], with stronger and sustained neural activity in response to natural human faces [12,13]. Furthermore, human characteristics, including mental states, are ascribed to a greater extent to more anthropomorphic representations of computer interfaces (e.g., [14]). In a study by Krach and colleagues [15], for instance, an anthropomorphic robot was considered more pleasant to interact with than a functional, machine-like robot and this led to enhanced cortical activity in brain regions implicated in the ToM network. The tendency to infer mental states thus seems to increase with the human realistic appearance of artificial entities.

Nonetheless, the presence of mind behind faces of varying realism is not continuous, but rather categorical. That is, faces are judged dichotomously as either alive, i.e., possessing mental states, or not [16,17]. In a recent study, Looser and Wheatley [16] demonstrated that on a continuum of faces modified from naturally ‘human’ to ‘artificial’ through a range of morphs between the two, faces that fell closer to the artificial endpoint were ascribed diminished levels of animacy and mental capacities of planning, feeling pain, and having a mind. Moreover, the point across the morphs’ continua at which faces were equally likely to be perceived as animate or inanimate—the point of subjective equality (PSE)—was found to be shifted towards the human endpoint. This suggested that attributions of animacy do not translate 1:1 onto the actual percentage of the humanness of a face, but instead are shaped by additional mechanisms involved in the perception of mind.

Having a mind is an important element of the concept of humanness. The self and one’s in-group are typically the best embodiments of the category of humans and are granted the full scope of human features (e.g., [18]). While the in-group is the source of positive identity and positive distinctiveness from other groups, out-group members are generally less favored and often viewed as less human than in-group members [19]. In particular, they are seen as lacking complex human feelings [20], as well as identity, status, and certain personality traits [21,22]. The failure to attribute traits that are unique or natural to all human beings may then reduce out-group members to animals or machines and deprive them of their moral value (see [23]).

Although dehumanization has been the focus of numerous studies that relied on descriptive representations of social groups (e.g., [24,25,26]), only little research so far used visual, and here specifically facial, stimuli (see [27,28]). One study by Hackel, Looser, and Bavel [29] recently explored the aspect of dementalization as evoked by faces and showed that group membership was one of the top-down motivations that shaped mind perception. Building on the findings by Looser and Wheatley [16], they had participants evaluate continua of facial morphs introduced as in-group or out-group members based on both minimal group assignment and labels of academic and political affiliation. Participants were found to apply stricter thresholds for seeing mind behind out-group faces and this bias was particularly pronounced for those who strongly identified with their in-group. The results provided first evidence that the degree of perceiving a mind in the other is contingent upon social dynamics, which thereby interact with basic perceptual processes. The question then arises to what extent the above effects generalize to other targets and perceivers.

### Aims of Present Research

Studies by Looser and Wheatley [16] and Hackel and colleagues [29], like the overwhelming majority of research on mind perception and dementalization, were conducted with predominantly White participants in a Western society (USA). This engenders the issue of cross-cultural generalizability of the findings. The aim of our research was therefore to extend previous work by contributing a cross-cultural comparison to the study of intergroup bias in perceptions of animacy and mind, conducted with a diversified sample of participants drawn from a Western (UK) and an Eastern (India) culture. We consider the continua of faces of varying realism to have a great potential as probes for social attitudes in an intergroup context. We further appropriated the original paradigm from Looser and Wheatley [16] in a way that the gradual change of faces on the morphs’ continua did not entail modifications to facial morphology.

To move beyond the use of the minimal group paradigm and category labels for social groups, as in Hackel and colleagues [29], we employed the faces to represent members of distinct ethnic groups. Racial or ethnic group membership is the most basic cue to rapid social categorization, besides age and gender, and has been a common foundation of research on prejudice. In many studies that used single faces or facial sequences of varying skin color (e.g., [30,31,32], participants’ responses to the facial stimuli were predicted by their levels of disposition (i.e., prejudice).

Given that mind perception necessitates the recognition of one’s moral status [33] and the motivation to grant moral regard to others is thought to be facilitated by empathy [34], we regarded people’s ability to share the feelings and perspectives of out-group members as an essential variable. This assumption is supported by evidence showing that out-groups evoke overall less empathic responses than in-groups and their suffering is less likely to be attended to [35,36]. In extreme cases, they may be even excluded from moral considerations (e.g., [37]). Therefore, instead of focusing on the role of prejudice in shaping perceptions of out-groups, our key variable in predicting judgments of animacy and mental capacities of out-group members was ethnocultural empathy. The construct’s operationalization is parallel to general empathy (e.g., [38]), except that it incorporates the aspect of cultural differences [39].

### Overview of Studies

In Experiment 1, Caucasian faces ranging from real to artificial were evaluated by participants in the UK and India on animacy, abilities to plan and to feel pain, and having a mind, in accord with Looser and Wheatley [16] and Hackel and colleagues [29]. Following the predictions provided by research on humanness and its denial to out-groups, we hypothesized that participants in the UK would perceive Caucasian (in-group) faces to be more alive and to experience mental states to a greater degree, compared to participants in India, for whom the same faces would represent an out-group. We also hypothesized that for participants in India the Caucasian (out-group) faces would need to look more human-like in order to be seen as human, with the PSE being shifted closer to the human endpoint of the morphs’ continua. Attributions of all characteristics should decrease as the faces approach the artificial endpoint of the continua. In Experiment 2, participants from India not only evaluated Caucasian (out-group) faces, but also South Asian (in-group) faces, with results predicted to closely mirror those of the UK participants judging Caucasian (in-group) faces in Experiment 1. For both studies, we hypothesized that individual differences in ethnocultural empathy would predict ratings of out-group, but not in-group, faces by participants in the UK and India.

## Experiment 1

The first experiment explored the process of out-group dementalization for Caucasian faces, whereby participants in the UK and India rated the faces ranging from real to artificial on animacy, abilities to plan and to feel pain, and having a mind.

### Method

#### Ethics statement

The experiment was conducted with ethics approval from the School of Psychology at Cardiff University, United Kingdom and the Department of Psychology, Karnatak University, India. All participants gave informed written consent before testing.

#### Participants

Thirty-three White British students at Cardiff University, United Kingdom (19 women; *M*_*age*_ = 22.15 years, *SD* = 5.15) and forty-four South Asian students at Karnatak University, India (22 women; *M*_*age*_ = 24.14 years, *SD* = 1.94) took part in the experiment on a voluntary basis. All participants completed the study in English, but were natives of the country in which they participated. None indicated familiarity with the stimuli. Informed consent was obtained prior to the experiment.

#### Stimulus material

**Fig 1:**
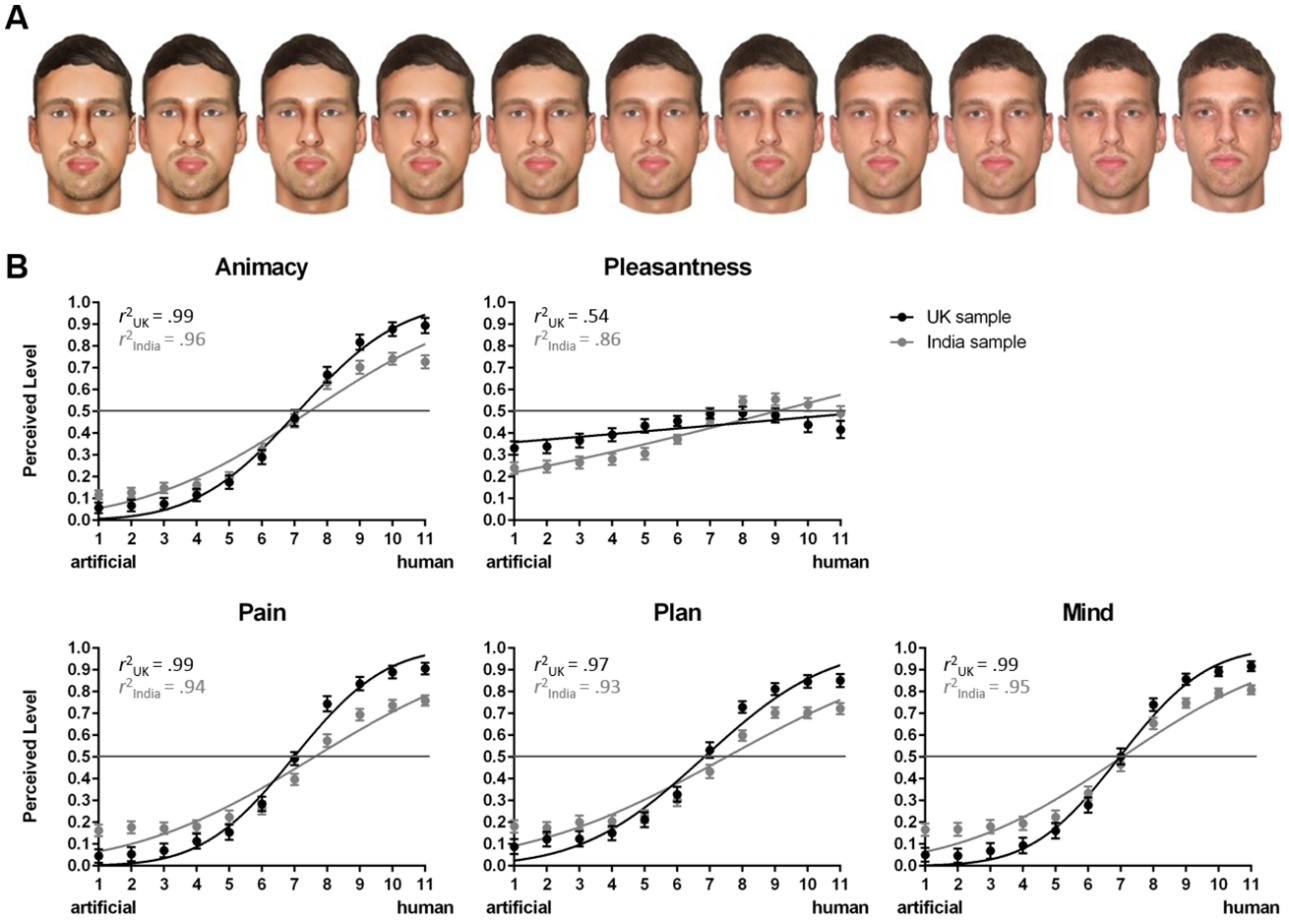
Stimulus example and results for Experiment 1. *A*. Example of a Caucasian target with 11 variants from artificial to human (right side) as used in Experiment 1. *B*. India and UK samples’ average ratings of Caucasian targets at each point along the morph continuum including error bars (SEM) and the fitted curves (solid lines) per measure. *r* ^2^ = model fit index.

Ten photographs of neutral faces of Caucasian men were selected from the Center for Vital Longevity Face Database [40]. They formed the human endpoint of the images’ continua. Modifications to the facial texture in each photograph were applied in Photoshop CS3 (Adobe Systems Inc., California, USA) by a professional graphics artist to create the faces’ artificial analogues, constituting the artificial endpoint. To obtain the intermediate images along the visual continuum, nine morphs with equal increments of physical change between every human face and its artificial equivalent were generated using FantaMorph 3.7.1 software (Abrosoft Co., Beijing, China). Thus, each of the 10 original faces had 11 variants (human, 9 morphs, artificial), which added up to 110 facial stimuli (10 faces x 11 variants). They were displayed in color on white background and measured 688 x 652 pixels (see Fig 1A).

#### Design and procedure

Stimulus evaluation was blocked for every measure, with all 110 images in each block. The experiment always started with evaluations of *animacy* and *pleasantness*. Ratings were made on 7-point Likert scales, with response options ranging from *1—definitely not alive* to 7—*definitely alive* for animacy, and 1—*very unpleasant* to 7—*very pleasant* for pleasantness. This was followed by evaluations of the faces’ abilities to feel *pain* and to formulate a *plan*, as well as whether the target possessed a *mind*. For these three questions the response options ranged from 1- *definitely not* to 7 –*definitely yes*. The ordering of the blocks was counterbalanced with the exception of *mind*, which was always presented last (see [16]). The facial stimuli within a block were shown in a random sequence and remained on the screen until a response was given. The experimental task was delivered by Python 2.7 (Python Software Foundation, Oregon, USA).

Participants were tested individually. After completion of the ratings, they filled out a paper-and-pencil version of the Scale of Ethnocultural Empathy [39]. It consists of 31 items targeting four dimensions of ethnocultural sensitivity: (a) Empathic feeling and expression, (b) Empathic perspective taking, (c) Acceptance of cultural differences, and (d) Empathic awareness. For all items response options ranged from 1 –*strongly disagree* to 6 –*strongly agree*.

### Results

#### Data preparation

Average scores were obtained across all facial exemplars for each of the 11 images of the morphs’ continua. In line with Looser and Wheatley [16], the resulting 11 image means were then linearly transformed from the original 7-point Likert scales into scores ranging from 0 to 1 (with higher scores indicating greater levels of the dimension). To obtain psychometric curves for the dependent measures, the standardized scores were fitted with a Gaussian distribution in GraphPad Prism 6 (GraphPad Software Inc., California, USA). This provided an overall fit index of participants’ judgment data to the mean estimated slope (see r^2^ in Fig 1B) and allowed the calculation of the Point of Subjective Equality (PSE). Outliers were identified on the individual PSE values for each participant falling beyond the *M* ± 2.5 *SD* range and were treated as missing data in the PSE analyses. For ratings of pleasantness, which did not fit a sigmoidal but a linear function (see also [16]), no PSE values could be calculated. Participants’ gender was excluded from all analyses due to lack of any significant effects of this variable in preliminary tests.

#### Analyses of variance

A multivariate repeated measures analysis of variance (RM-MANOVA) with Participant Sample (UK, India) as a between-subjects factor and Morph Level (1–11) as a within-subjects factor was performed on the standardized scores of *animacy*, *pleasantness*, *pain*, *plan*, and *mind*. For all univariate analyses, a Greenhouse-Geisser adjustment to degrees of freedom was applied and Sidak correction was used for multiple comparisons.

Significant multivariate effects emerged for Morph Level, *F*(50, 3700) = 17.65, *p* < .001, η_p_^2^ = .19, and Morph Level × Participant Sample, *F*(50, 3700) = 5.00, *p* < .001, η_p_^2^ = .06. In univariate terms, the interaction between Morph Level and Participant Sample was significant for all measures of interest: *animacy*, *F*(1.92, 74) = 6.06, *p* = .003, η_p_^2^ = .08; *pleasantness*, *F*(1.51, 74) = 6.36, *p* = .005, η_p_^2^ = .08; *plan*, *F*(1.63, 74) = 6.43, *p* = .004, η_p_^2^ = .08; *pain*, *F*(1.73, 74) = 11.41, *p* < .001, η_p_^2^ = .13; and *mind*, *F*(2.01, 74) = 8.60, *p* < .001, η_p_^2^ = .10.

Pairwise comparisons showed that participants in India perceived the facial images located towards the artificial end of the morphs’ continua as more *alive* (morph 3, *p* = .044), more able to formulate a *plan* (morph 1, *p* = .033) and to feel *pain* (morphs 1–3, *p*s < .01), and more likely to possess a *mind* (morphs 1–4, *p*s < .05) than the UK sample. The inverse was true for the opposite end of the continua. Here, participants in the UK rated the images belonging to the human end as more *alive* (morphs 9–11, *p*s < .05), more capable of formulating a *plan* (morphs 8–11, *p*s < .01) and feeling *pain* (morphs 7–11, *p*s < .05), and more likely to possess a *mind* (morphs 8–11, *p*s < .05) than participants in India. Interestingly, participants in the UK also rated the faces along the artificial half to be more *pleasant* than did the Indian participants (morphs 1–6, *p*s < .05) (see Fig 1B).

#### Point of Subjective Equality (PSE) analyses

For each sample (UK, India), PSE values were derived from the fitted curves in GraphPad Prism 6 where the face ratings correspond to the ordinate midpoint of a measure (horizontal line at value 0.5 in figures). In accordance with Looser and Wheatley’s [16] reasoning, if perceptions of animacy are presumed to linearly map onto the percentage of the human face in every morph, then the point at which the face is equally likely to be judged as animate or inanimate should correspond to morph 6 on the abscissa where the face is half animate/inanimate (50%). As can be seen in Table 1, considerable deviations of the PSE values from the morphs’ continua midpoint were observed for both samples on *animacy*, *plan* and *pain*, *t*s > 2.0, *p*s ≤ .05, and for the UK sample on *mind*, *t*(32) = 4.10, *p* < .001, indicating a shift in perception thresholds towards the human endpoint.

**Table 1:** Mean PSE Values per Measure in Experiment 1.

	UK sample	India sample
Measure	*M (SD)*	*M (SD)*
**Animacy**	6.96 (1.51)	7.28 (1.86)
**Pain**	7.06 (1.23)	7.63 (1.16)
**Plan**	6.81 (1.09)	8.09 (1.98)
**Mind**	6.83 (1.17)	7.26 (1.72)

*Note*. Mean PSE values are given in terms of the original morph numbers (1–11).

To test for significant differences in PSE values between the UK and Indian sample, a multivariate analysis of variance (MANOVA) with Participant Sample (India, UK) as a between-subjects factor was performed on the PSE scores for *animacy*, *pain*, *plan*, and *mind*. As predicted, the multivariate main effect was significant for Participant Sample, *F*(4, 59) = 3.93, *p* = .007, η_p_^2^ = .21, with participants in India generally displaying higher thresholds for attributing human traits to Caucasian faces than participants in the UK. On the univariate level, this difference was statistically significant for *plan*, *F*(1, 62) = 10.34, *p* < .001, η_p_^2^ = .14, and marginally significant for *pain*, *F*(1, 62) = 3.64, *p* = .061, η_p_^2^ = .06.

#### Regressions

Regression analyses were performed to test whether the degree of ethnocultural empathy of participants in the UK and India significantly predicted the PSE values for the four measures. Results revealed that only for the Indian sample the PSE value for *animacy* was significantly predicted by their overall ethnocultural empathy score, β = ˗.34, *t*(39) = ˗2.24, *p* = .031. In order to demonstrate that the effect of ethnocultural empathy was specific to Indian participants viewing Caucasian faces, we submitted participants’ PSE scores of animacy to a Participant Sample (1 = India, -1 = UK) × Ethnocultural Empathy multiple regression analysis. Due to the bimodal distribution of the ethnocultural empathy data (with peaks at values 110 and 135) a median split (127.5) was performed on the variable, thereby revealing the hypothesized interaction with Participant Sample when entered as a categorical variable in the multiple regression analysis, β = 1.18, *t*(71) = 2.49, *p* = .015. Specifically, Indian participants displayed elevated thresholds for perceiving Caucasian faces as *alive* when they scored low rather than high on ethnocultural empathy (*M*_low_ = 8.89 vs. *M*_high_ = 6.33, *p* = .001). For participants in the UK, PSE values for animacy did not significantly vary with the degree of ethnocultural empathy (*M*_low_ = 6.91 vs. *M*_high_ = 6.99, *p* = .922).

### Discussion

Participants in the UK generally perceived the faces as more *alive*, more likely to possess a *mind*, and more able to feel *pain* and to formulate a *plan* than participants in India. At the same time, participants in India evaluated the faces towards the artificial endpoint of the morphs’ continua higher on these traits than participants in the UK. These results suggest that participants in the UK, who viewed the in-group faces and judged them to be more human-like than participants in India, favored the in-group in the attribution of ToM qualities. They were more conservative than participants in India in attributing those qualities when the faces became more artificial.

In line with Looser and Wheatley [16] and Hackel and colleagues [29], the PSEs shifted towards the human endpoint of the morphs’ continua. When comparing the two samples, the Indian participants’ PSE for *plan* and *pain* was closer to the human endpoint than that of UK participants, suggesting subtle out-group dehumanization as Caucasian faces needed to look more human-like in order for them to be seen as able to formulate a *plan* and feel *pain*. Furthermore, the PSE for *animacy* was significantly predicted for the Indian but not for the UK sample by the degree of ethnocultural empathy, indicating the involvement of ethnocultural sensitivity in the perception of out-group, but not in-group, faces.

## Experiment 2

The purpose of the second experiment was to investigate how participants in India evaluated South Asian faces, representing their in-group, and Caucasian faces with respect to animacy, abilities to plan and to feel pain, and having a mind.

### Method

#### Ethics statement

The experiment was conducted with ethics approval from the Department of Psychology, Karnatak University, India. All participants gave informed written consent before testing.

#### Participants

Sixty-six young adults (29 women), age range 21–31 years (*M* = 23.29, *SD* = 2.01) at Karnatak University, India, volunteered to take part in Experiment 2. All participants completed the study in English, but were natives of India. None had participated in the previous study and indicated familiarity with the stimuli. Informed consent was obtained prior to participation.

#### Stimulus material

**Fig 2:**
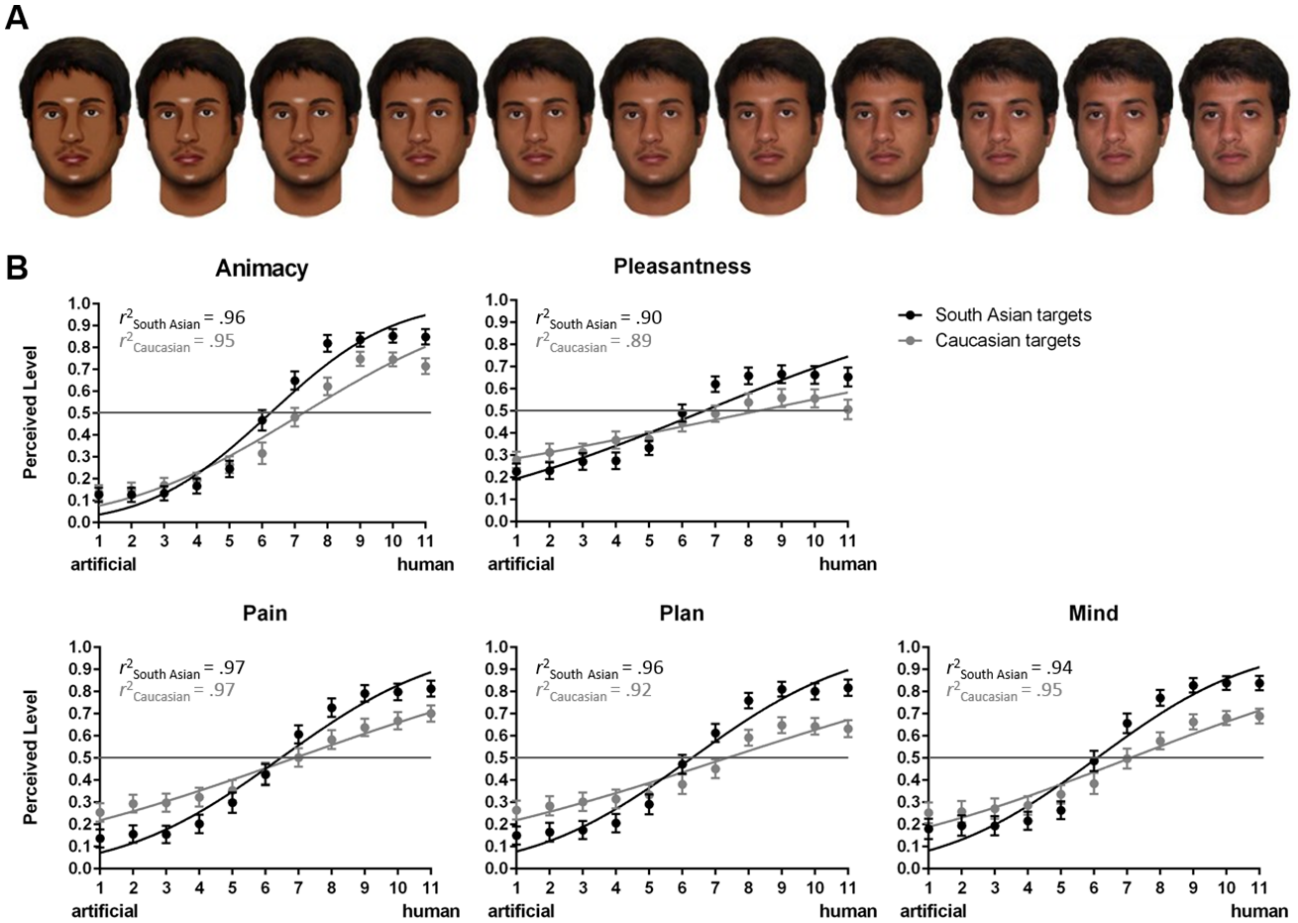
Stimulus example and results for Experiment 2. *A*. Example of a South Asian target with 11 variants from artificial to human (right side) as used in Experiment 2. *B*. Indian participants’ average ratings of South Asian and Caucasian targets at each point along the morph continuum including error bars (SEM) and the fitted curves (solid lines) per measure. *r* ^2^ = model fit index.

In addition to the Caucasian faces from Experiment 1, ten photographs of neutral faces of South Asian men were selected from the Center for Vital Longevity Face Database [40] to depict the human endpoint of the morphs’ continua. The faces matched those of the Caucasian targets considering attractiveness (*M* = 1.9), intelligence (*M* = 2.77), trustworthiness (*M* = 2.55), and likeability (*M* = 2.67; scale 1–5, all *p*s > .05), as determined in a pilot study (*n* = 30). The faces’ artificial analogues were generated in Photoshop CS3 (Adobe Systems Inc., California, USA) in the same way as in Experiment 1 and morphed with the human photographs in FantaMorph 3.7.1 software (Abrosoft Co., Beijing, China) to create the 11 variants of each of the 10 target faces. The final set consisting of 110 South Asian and 110 Caucasian facial stimuli was displayed in color on white background and measured 688 x 652 pixels (see Fig 2A for an example of a South Asian face).

#### Design and procedure

Design and procedure were identical to Experiment 1. Participants were randomly assigned to view either South Asian or Caucasian faces.

### Results

The data preparation steps followed exactly those in Experiment 1. The model fit values (r^2^) are indicated in Fig 2B.

#### Analyses of variance

A RM-MANOVA with Target Ethnicity (South Asian, Caucasian) as a between-subjects factor and Morph Level (1–11) as a within-subjects factor was performed on the standardized scores of *animacy*, *pleasantness*, *pain*, *plan*, and *mind*. For all univariate analyses, a Greenhouse-Geisser adjustment to degrees of freedom was applied and Sidak correction was used for multiple comparisons.

Significant multivariate effects emerged for Morph Level, *F*(50, 3200) = 12.50, *p* < .001, η_p_^2^ = .16, and Morph Level × Target Ethnicity, *F*(50, 3200) = 2.30, *p* < .001, η_p_^2^ = .04. In univariate terms, the interaction between Morph Level and Target Ethnicity was significant for all measures: *animacy*, *F*(2.24, 64) = 4.22, *p* = .013, η_p_^2^ = .06; *pleasantness*, *F*(1.71, 64) = 4.38, *p* = .020, η_p_^2^ = .06; *plan*, *F*(2.03, 64) = 9.16, *p* < .001, η_p_^2^ = .13; *pain*, *F*(2.00, 64) = 6.49, *p* = .002, η_p_^2^ = .09; and *mind*, *F*(2.05, 64) = 6.59, *p* = .002, η_p_^2^ = .09.

Pairwise comparisons showed that Caucasian faces were rated towards the artificial end of the morphs’ continua as more able to formulate a *plan* (morph 3, *p* = .034) and to feel *pain* (morphs 1–4, *p*s < .05) than South Asian faces. The inverse was true for the human end of the continua. Here, South Asian Faces were rated as more *alive* (morphs 6–8 and 10–11, *p*s < .05), more *pleasant* (morphs 7–8 and 11, *p*s < .05), more capable of formulating a *plan* (morphs 7–11, *p*s < .01) and feeling *pain* (morphs 8–11, *p*s < .05), and more likely to have a *mind* (morphs 7–11, *p*s < .05) compared to Caucasian faces (see Fig 2B).

#### PSE analyses

Due to violations of the assumption of homogeneity of variance, differences in PSE scores from the morphs’ continua midpoint (morph 6) were analyzed using a nonparametric one-sample Wilcoxon signed rank test. As can be seen in Table 2, significant deviations of the PSE values from the midpoint were observed for both types of faces on *pain*, *Z*s > 2.2, *p*s < .05, and for Caucasian faces on *animacy*, *Z* = 2.75, *p* = .006; *plan*, *Z* = 2.50, *p* = .012; and *mind*, *Z* = 2.14, *p* = .032, suggesting that perception thresholds were shifted towards the human endpoint.

**Table 2:** Mean PSE Values per Measure in Experiment 2.

	South Asian targets	Caucasian targets
Measure	*M (SD)*	*M (SD)*
**Animacy**	6.58 (2.30)	7.88 (2.63)
**Pain**	6.59 (1.80)	8.02 (3.18)
**Plan**	6.37 (1.17)	7.82 (3.15)
**Mind**	6.02 (2.61)	7.94 (3.44)

*Note*. Mean PSE values are given in terms of the original morph numbers (1–11).

In order to test for significant differences in PSE values between South Asian and Caucasian faces, a MANOVA with Target Ethnicity (South Asian, Caucasian) as a between-subjects factor was performed on the log-transformed PSE scores for *animacy*, *pain*, *plan*, and *mind*. As predicted, the main effect of Target Ethnicity was significant on the multivariate level, *F*(4, 40) = 5.97, *p* = .001, η_p_^2^ = .37. The same applied to all measures on the univariate level, *animacy*, *F*(1, 43) = 9.27, *p* = .004, η_p_^2^ = .18; *plan*, *F*(1, 43) = 18.20, *p* < .001, η_p_^2^ = .30; *pain*, *F*(1, 43) = 6.73, *p* = .013, η_p_^2^ = .14; and *mind*, *F*(1, 43) = 18.10, *p* < .001, η_p_^2^ = .30, with higher thresholds for perceiving human traits in Caucasian faces compared to South Asian faces.

#### Regressions

Regressions analyses showed that Indian participants’ ethnocultural empathy score was a marginal significant predictor of the PSE value for *animacy* (log-transformed) only for Caucasian targets, β = ˗.37, *t*(27) = ˗2.02, *p* = .054. In order to demonstrate that the effect of ethnocultural empathy was specific to Indian participants viewing Caucasian faces, we submitted participants’ log-transformed PSE scores of animacy to a Target Ethnicity (1 = Caucasian target, -1 = South Asian target) × Ethnocultural Empathy multiple regression analysis. Due to the bimodal distribution of the ethnocultural empathy data (with peaks at values 120 and 140) a median split (128.0) was performed on the variable, thereby revealing the hypothesized interaction with Target Ethnicity when entered as a categorical variable in the multiple regression analysis, β = -.81, *t*(57) = -2.01, *p* = .049. Specifically, Indian participants displayed elevated thresholds for perceiving Caucasian faces as *alive* when they scored low rather than high on ethnocultural empathy (*LogM*_low_ = .96 vs. *LogM*_high_ = .77, *p* = .003). This was not the case when evaluating South Asian faces for which thresholds of animacy did not significantly vary as a function of ethnocultural empathy (*LogM*_low_ = .79 vs. *LogM*_high_ = .77, *p* = .774).

### Discussion

This experiment demonstrated that participants in India perceived the South Asian (in-group) faces as more *alive*, more *pleasant*, better able to formulate a *plan* and to feel *pain*, as well as more likely to possess a *mind* than the Caucasian (out-group) faces. These observations correspond to the in-group favoritism in the attribution of ToM qualities found in Experiment 1. Participants were also more conservative in attributing *plan* and *pain* to South Asian faces when these became artificial. In addition, the present data validated the effect of out-group dehumanization. This was shown by the PSE values falling closer to the human endpoint of the morphs’ continua for Caucasian compared to South Asian faces, suggesting that out-group faces needed to be more human-like in order to be considered by participants in India as possessing human traits. Replicating the findings from the first study, ethnocultural empathy emerged again as a significant predictor of the PSE for animacy in ratings of out-group, but not in-group, faces.

## General Discussion

The main goal of our research was to examine whether intergroup biases in mind perception generalize across cultures. Whilst previous work has employed minimal and academic/ political affiliation groups [29], we pursued the topic of mind perception in a context where participants from both Western (UK) and Eastern (India) cultural backgrounds assessed ethnically diversified faces that represented their respective in-group and out-group. In Experiment 1, the results showed that participants in the UK perceived the Caucasian faces towards the human endpoint of the morphs’ continua to be more alive, more able to formulate a plan and to feel pain, and more likely to possess a mind than participants in India. Participants in India ascribed these traits to a greater degree to the Caucasian faces situated towards the artificial endpoint of the morphs’ continua. Furthermore, they judged the PSE for plan and pain to fall closer to the human endpoint than participants in the UK. In Experiment 2, the results closely mirrored those of the first study in that participants in India perceived the South Asian faces as being more alive, more able to plan and feel pain, and more likely to have a mind than Caucasian faces, and this was again true for the human endpoint of the morphs’ continua.

Together, these findings extend those by Looser and Wheatley [16] and Hackel and colleagues [29] by revealing an in-group bias in the perception of ToM characteristics for participants in the UK as well as in India. Moreover, evidence was found for subtle out-group derogation. The differences in the PSE were indicative of out-group dementalization for participants in India, whereby Caucasian faces had to look more human-like than South Asian faces in order to be recognized as possessing human traits. To our knowledge, this is the first study that employed in-group and out-group faces with varying levels of artificiality to investigate intergroup attitudes in a cross-cultural context. Ethnicity as a basis for the division into an in-group and an out-group served as an ecologically valid cue for group membership and went beyond the widely studied racial categories (i.e., Blacks/Whites, see [41] for a review).

Participants’ tendency to attribute animacy and mental capacities to a greater degree to the faces positioned closer to the human endpoint of the morphs’ continua may be interpreted as an attempt to reserve these qualities only for faces that could be unquestionably categorized as belonging to the respective in-groups. As the faces progressively became artificial, an ambiguity as to whether they still represented the in-group members might have arisen. Such increasing artificiality consequently made participants more conservative in attributing human-like traits to in-group members. This paradoxical effect is consistent with the in-group over-exclusion phenomenon [42,43]. It is manifested in a bias to accept fewer individuals into the in-group than to the out-group, which shields the former from erroneous inclusion of those who do not belong and thus enables the in-group to maintain integrity. The protection of the in-group’s humanness from contamination with nonhuman (e.g., animal-like) features has been found previously in the context of cultural groups (Northern and Southern Italians; [44]). Our findings may suggest a corresponding effect for the protection of the in-group’s mind from contamination with object-like, mindless artificial entities.

The evaluations of the other’s humanness further turned out to be shaped by the subjectivity of the perceiver, that is, by the level of ethnocultural empathy. Ethnocultural empathy was a significant predictor of the PSE values for animacy when the target faces represented the out-group. No such effect occurred for the in-group faces. This highlights the impact of dispositional variables on basic perceptual processes, particularly on categorization according to one of the most fundamental distinctions between the animate and inanimate (e.g., [45]). The question of what is alive and what is not therefore seems to rely less on objective criteria (e.g., realism) applied to the target. Instead, the variation in judgments of life depending on one’s group membership indicates that the “tipping point of animacy” (p. 1854, [16]) may be determined by what the face stands for on a higher, i.e., group, level. Presently, empathy towards cultural/ethnic groups was found to act as a driving force in intergroup perception and to mitigate its negative aspect of out-group dementalization [46]. Interestingly, this was the case for attributions of animacy, but not for other variables such as pain, plan or mind. Future research might want to follow up on those specific findings, thereby making an explicit distinction between agency and experience related traits as two components of mind perception when exploring the role of ethnocultural empathy [29,33].

Empathy is viewed as a skill that can be trained and engaged to foster positive out-group attitudes and improve intergroup relations [47,48]. Accordingly, ethnocultural empathy appears to be a proper instrument for combating biases that automatically emerge at an early stage of human perception and determine later derogative responses. Interventions designed to boost empathy towards out-groups often concentrate on the development of the perspective taking ability, which is an important constituent of cultural-specific empathy and has been shown to weaken intergroup prejudice [49,50]. Contemporary empathy training turns to artificial characters to symbolize out-group members in virtual realities and demonstrates that identification with such entities, achieved through the experience of ownership of another’s body or body parts, successfully decreases implicit racial bias [51,52].

Despite the fact that the realism of computer-generated entities may be sufficient for the user to establish an effective connection with them, the creation of highly human-like characters, and especially of photorealistic faces, remains one of the “ultimate challenges” in the fields of computer graphics and animation (e.g., [6]). Major efforts have been devoted to perfecting the potentially problematic elements of faces, but this was typically done as if these were a collection of separate features, colors, and textures (e.g., [53,54]). Nevertheless, in the end even a very authentic face may be imbued with less human qualities and consequently elicit less social reactions if it represents an out-group member. Throughout the paper, we showed that perceptions of humanness stem from what the face stands for as a social construct and we underlined that group membership plays a central role in how alive it appears and to what extent mental capacities are attributed to it.

## Supporting Information

S1 Dataset Supporting data for Experiment 1 and 2. (ZIP)
